# A Case of Late Tetanus

**Published:** 1918-09

**Authors:** 


***A Case of Late Tetanus.*** By EDWARD ANTOINE. *La Presse Médicale,* June 10, 1918.

The writer gives full particulars of an extraordinary case of late tetanus. There was a fracture of the right thigh complicated with gas gangrene. The patient could not be removed from the field for 30 hours, and the first dose of antitetanic serum could not be given until the time of removal. A second dose was given eight days later. Both doses were small (10 cc. each).

The wound healed and the patient started to walk. On the fourth or fifth day later (78th day after injury and the 60th day after the expiration of the immunity from the serum) tetanus set in.

The writer calls attention to the following peculiarities of the case:

1. The rarity of late tetanus after a second dose of antitetanic serum.

2. The failure of the tetanus to break out immediately after the expiration of immunity.

3. The probability that the organisms were encysted and set at liberty by the tearing of the newly formed tissues in the first effort to walk.

4. The combination of what apparently was both delayed and secondary tetanus, that is, tetanus postponed by the period of immunity and then brought out by a subsequent traumatism.

5. The localization of the first symptoms in the right leg and the later generalization in an attenuated form of the usual tetanic phenomena — trismus, collapse, respiratory disorders, — all of which symptoms disappeared in the course of 8 or 10 days, apparently as a result of rigorous treatment. The evolution was gradual and the local rigidity in the wounded limb persisted for about five weeks.

6. Attempts to stop treatment were followed by relapses or aggravation of the symptoms.

The author emphasizes the fact that stands out strongly in the case, namely, that *any definite traumatism is sufficient to bring to life a dormant tetanic infection, especially if the patient is not sufficiently immunized.* He thinks the case proves the efficacy of prophylactic injections, but he believes they should be given in doses of from 15 to 20 cc. He recommends the greatest prudence in allowing the seriously wounded, especially in case of open fractures, to walk or even to leave the bed until a period later than 60 days. He even believes a dose of antitetanic serum would be justified in certain cases before such an attempt.

He urges by way of treatment not only large doses of serum but also subcutaneous injections of huile phéniquée, carbolic oil, at the root of the injured limb and in the region of the fracture. He attributes the success of his treatment in this case to the persistent use of these injections.

